# Genetic Heterogeneity in Algerian Human Populations

**DOI:** 10.1371/journal.pone.0138453

**Published:** 2015-09-24

**Authors:** Asmahan Bekada, Lara R. Arauna, Tahria Deba, Francesc Calafell, Soraya Benhamamouch, David Comas

**Affiliations:** 1 Département de Biotechnologie, Faculté des Sciences de la Nature et de la Vie, Université Oran 1 (Ahmad Ben Bella), Oran, Algeria; 2 Institut de Biologia Evolutiva (CSIC-UPF), Departament de Ciències Experimentals i de la Salut, Universitat Pompeu Fabra, Doctor Aiguader 88, 08003 Barcelona, Spain; 3 Centre de Transfusion Sanguine- Centre Hospitalo-Universitaire d’Oran (CTS-CHUO), Oran, Algeria; Erasmus University Medical Center, NETHERLANDS

## Abstract

The demographic history of human populations in North Africa has been characterized by complex processes of admixture and isolation that have modeled its current gene pool. Diverse genetic ancestral components with different origins (autochthonous, European, Middle Eastern, and sub-Saharan) and genetic heterogeneity in the region have been described. In this complex genetic landscape, Algeria, the largest country in Africa, has been poorly covered, with most of the studies using a single Algerian sample. In order to evaluate the genetic heterogeneity of Algeria, Y-chromosome, mtDNA and autosomal genome-wide makers have been analyzed in several Berber- and Arab-speaking groups. Our results show that the genetic heterogeneity found in Algeria is not correlated with geography or linguistics, challenging the idea of Berber groups being genetically isolated and Arab groups open to gene flow. In addition, we have found that external sources of gene flow into North Africa have been carried more often by females than males, while the North African autochthonous component is more frequent in paternally transmitted genome regions. Our results highlight the different demographic history revealed by different markers and urge to be cautious when deriving general conclusions from partial genomic information or from single samples as representatives of the total population of a region.

## Introduction

The human history of North Africa has been shown to be a complex demographic process characterized by multiple migrations, admixtures and founder effects. It has been suggested that the first occupation of the area by modern humans, attested by the Aterian culture, might be dated back to ~160,000 years ago [1]; and posterior cultures have been imposed in the region during pre-Holocene and Holocene times [2]. Despite the long-standing presence of human cultures in the region, it has been suggested that the present-day populations in North Africa are the result of a recent back-to-Africa migration in pre-Holocene times that replaced the first inhabitants in the region, followed by multiple migrations from neighboring areas [3]. One of the most relevant human groups in the area are Berbers who are supposed to be the descendants of this first migration back-to-Africa from the Middle East; however, the dynamics of human groups living in that area is still unclear. Historical events testify of many invasions, conquests and migrations by Phoenicians, Romans, Vandals, Byzantines, Jews, Spanish, and French [4], as well as the presence of autochthonous groups such as the Libyans, Moors, Gaetuli, and Numidians, among others. However, the most important event was the Arab conquest that begun during the 7th century, when North-African autochthonous Berber populations were converted to Islam and since then Arabic has became official language employed in the region. This fact influenced the geographical distribution of Berber communities, which are nowadays relegated to peripheral and relict areas in a vast region extending from Mauritania to Egypt and from the Sahara desert to the Algerian and Moroccan Atlas mountainous areas [5]. Nomadism is one of the factors that have contributed to the geographic isolation of these Berber populations, which became slightly different in their dialect languages and cultures. Therefore, the North African population represents a mosaic of peoples at different levels: the spoken language, the culture and even the social organization that shows in the split observed between the urban regions representatives of the elite (Romanized and Arabicized populations, for example) and the Berber populations living in the rural areas.

The genetic characterization of North African populations has been carried out with the analysis of uniparental (mitochondrial, mtDNA; and Y-chromosome) and autosomal markers [3,6–17]. Beyond subtle differences in their conclusions, most analyses agree on i) the complex demographic pattern of migrations in the region suggesting a North African autochthonous component (represented for instance by the presence of U6 mtDNA and E1b1b1b-M81 Y-chromosome haplogroups); ii) an influence from surrounding areas through migrations from the Middle East, Europe, and sub-Saharan Africa; iii) sexual bias in the ancestral component admixture; and iv) extensive genetic drift due to bottlenecks and inbreeding.

Within the North African context, Algeria represents a key point to understand the human population history of the region. Located in the fertile coastal plain of North Africa, Algeria shares borders with Morocco, Western Sahara, Mauritania, Mali, Niger, Libya, and Tunisia. Taken together, Algeria, Morocco, and Tunisia form what is known as the Arab Maghreb. Algeria is now Africa's largest country, covering an area of nearly 2.4 million km^2^; of which 80% is occupied by the Algerian Sahara. On this vast territory, which extends from the Mediterranean environment in the north to the Saharan desert in the South, various human groups use the same language, the Berber, a generic name for any spoken Amazigh dialects. In fact, there are various Berber dialects spoken by different Berber groups in Algeria: Chaoui, Kabyle, M'zab, Zenete, Chleuh, and Touareg. Despite the human diversity described in Algeria and its geographical extent in the area, the knowledge about the human genetic diversity in the country is relatively scarce. A few studies have focused on a single Algerian sample in the area [3,8–10,18] extrapolating their results to a vast geographic area and ignoring the putative internal genetic diversity and heterogeneity in the region. In addition, the whole of North Africa is represented in the HGDP panel by a single Algerian Berber population, the Mozabites ([19]; and all the studies using the HGDP panel).

In order to overcome these limitations and to explore the genetic heterogeneity within Algeria, the present study describes the distribution of the uniparental lineages (mtDNA and Y-chromosome) and whole genome autosomal SNPs in several Algerian samples, including Arab- and Berber-speaking groups.

## Material and Methods

### Ethic statements

Written informed consent was obtained from the participants and analyses were performed anonymously. The present project obtained the ethics approval from the local Institutional Review Board, Comitè Ètic d’Investigació Clínica–Institut Municipal d’Assistència Sanitària (CEIC-IMAS) in Spain (2013/5429/I), as well as the approval from the local committee CRASC (Centre de Recherche en Antrhopologie Sociale et Culturelle) in Oran, Algeria.

### Subjects and Populations

**Fig 1:**
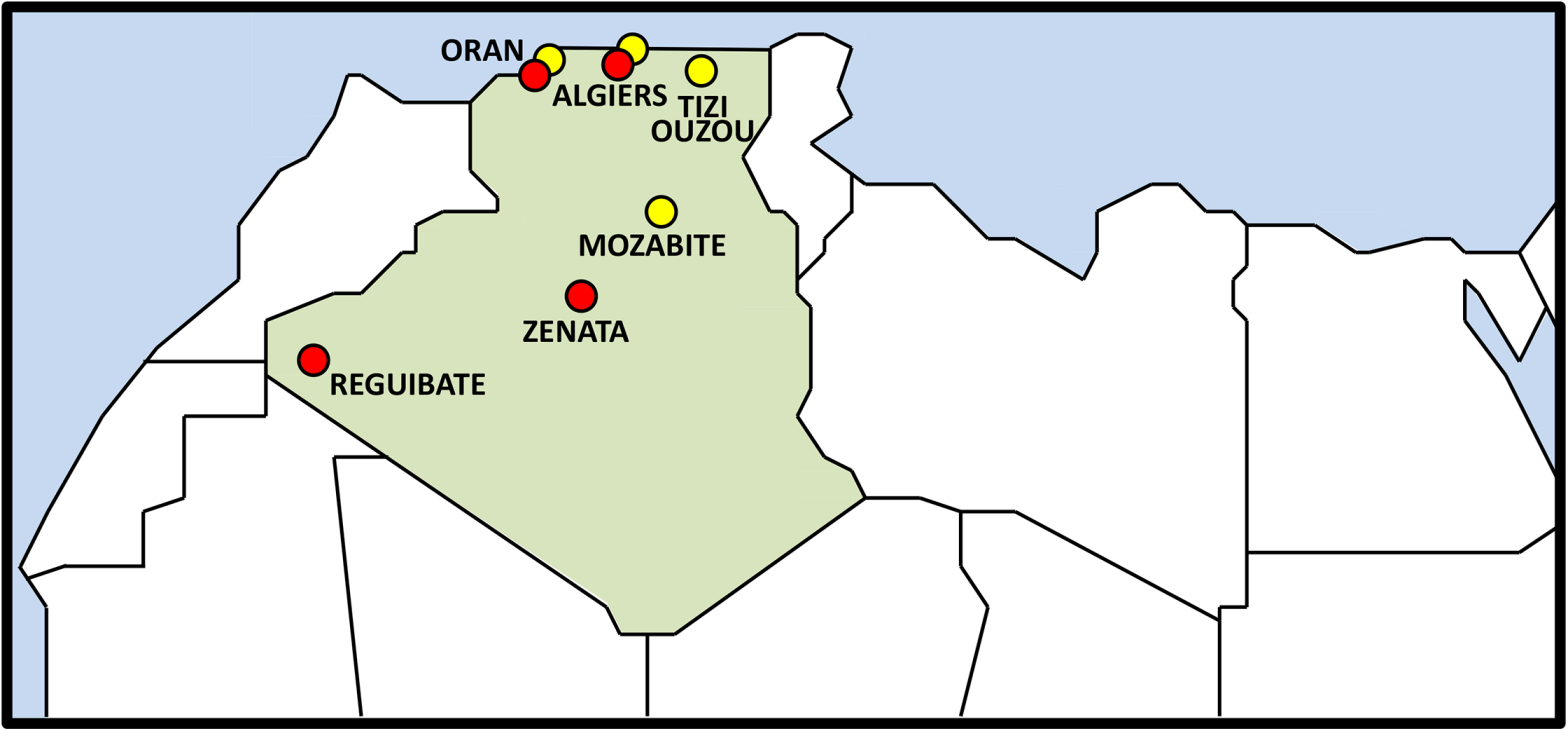
Geographic location of the Algerian samples genotyped in the present study (in red) and the samples obtained from the literature (in yellow).

Four populations from Algeria were genotyped in this study (Oran, Algiers, Reguibate and Zenata) (Fig 1). The sampling from Algiers and Oran, the largest cities in Algeria, was performed on the general population. In Oran city, volunteers were blood donors at the blood transfusion center of the University Hospital of Oran (CTS-CHUO). The Reguibate population was founded by Sidi Ahmed al-Rgibi (also known as Er Regubi) who lived in the Saguia el-Hamra region (in the north part of Western Sahara) in the 16th century. They were originally a nomadic tribe but nowadays they are settled in Morocco, Western Sahara and Mauritania, as well as in Algeria, in the region of Tindouf (in southwest Algeria close to the Mauritanian, Saharawi and Moroccan borders) where the sample was obtained. All the sampled individuals speak Hassaniya Arabic (a dialect very close to the literary Arab). The Zenata population, also called Zenet or Iznaten, is an ethnic Berber group in North Africa that is spread from Libya to Morocco. They speak a Berber dialect called Zenet or Zetani, which have some similitude with other Berber dialects. The Zenata individuals sampled are residents in the city of Timimoun, a little oasis village in Adrar Province, in the Gourara region (West Algerian Sahara).

Blood samples were anonymously collected from unrelated healthy adult volunteers and an informed consent was systematically signed by the individuals having understood all the necessary information concerning the project and agreed to be included in this study. The socio-demographic data, including genealogical and spoken dialect information, were collected from a questionnaire beforehand established. DNA was extracted using the standard salting-out method [20] and then quantified using the NanoDrop 2000 spectrophotometer (Thermo scientific). For Y-chromosome analysis, a total of 201 male samples were genotyped: 26 from Algiers, 80 from Oran, 60 Reguibate, and 35 Zenata. Additional published samples from Oran (n = 102) [17,21], Algiers (n = 35) [10], Tizi Ouzou (n = 19) [10], and 20 Mozabites from the city of Ghardaïa [22] were included in this study for comparative purposes. For mitochondrial DNA analysis, a total of 336 DNA samples were genotyped, (62 Algiers, 93 Oran, 108 Reguibate, 73 Zenata), and 240 individuals from Oran [17] and 85 Mozabites [18] were included in the comparative analyses.

### Uniparental marker analysis

Samples were genotyped for seventeen Y-chromosome STRs using the Amp*l*STR® Yfiler® PCR Amplification Kit (Applied Biosystems) according to manufacturer’s instructions. DNA fragments were separated and detected in an ABI Prism 3130 Genetic Analyzer (Applied Biosystems). Two additional Y-STRs (DYS426, DYS388) were typed using a PCR multiplex including 6 Y-SNPs (M17, M175, M60, M186, M91, and M139) [23] (S1 Table). Further 30 Y-chromosome SNPs were genotyped using TaqMan® probes (Applied Biosystems) [23]. The Real-time PCR was performed using ABI PRISM® 7900HT Sequence Detection System (Applied Biosystems). Haplogroups were further subdivided by genotyping 129 Y-chromosomal SNPs with the Open Array platform on 44 samples [24] and analyzed with AutoCaller™ Software 1.1 (Applied Biosystems, Inc.). Haplogroups were formally designated according to Karafet et al. [25], and refined following the International Society of Genetic Genealogy website (http://www.isog/tree).

The hypervariable segment I (HVSI) of mitochondrial DNA (mtDNA) was sequenced. The mtDNA control region was PCR amplified using primers pairs H408/L15996 [26]. Successfully amplified products were sequenced for complementary strands using primers L15996/H16401 and the Big Dye Terminator v3.1 Cycle Sequencing kit (Applied Biosystems). Samples were run on the ABI Prism 3130xl Genetic Analyzer (Applied Biosystems), according to the manufacturer’s instructions. Variable positions throughout the control region were determined from positions 16024–16391 according to the Cambridge Reference Sequence (CRS) [27]. For major haplogroup assignation (L3/L4, N, R, M), four TaqMan probes (Applied Biosystems) were used, following supplier’s recommendations, to genotype positions 3594, 10873, 12705, and 14783 [13]. In order to refine the haplogroup assignation of some samples, 17 coding region SNPs were genotyped using a multiplex SNaPshot assay (GenoCoRe22) [28]. Samples belonging to the H haplogroup were further subdivided using SNaPshot reactions (for H3 and H13 subgroups) [13], and PCR-RFLPs (for H1 and H3 subgroups) [29]. The mtDNA sub-haplogroups was performed using nomenclature proposed by van Oven and Kayser [30] and updated in www.phylotree.org (mtDNA tree Build 15; 30-9-2012) Y-chromosome and mtDNA haplogroup and haplotype frequencies and diversities were calculated using Arlequin software version 3.5 [31], as well as pairwise R_ST_ and F_ST_ distances between populations and their significance assessed by a nonparametric permutation test. Since Y-chromosome and mtDNA haplogroup resolution was not uniform across studies, the haplogroups were reduced to the most informative derived shared markers. Principal component analyses (PCA) were performed using IBM SPSS Statistic 19 version (SPSS Inc., Chicago, Illinois). Furthermore, the mtDNA sequence comparison was carried out on HVSI positions from 16024 to 16383, and 10 shared Y- chromosomal STRs (DYS389I, DYS390, DYS389II, DYS19, DYS393, DYS391, DYS439, DYS392, DYS437, and DYS438) were considered to study the haplotype diversity and to perform the non-metric multidimensional scaling (MDS) by IBM SPSS statistics 19 (SPSS Inc., Chicago, Illinois) using R_ST_ and F_ST_ distances between populations. MDS co-ordinates were plotted with R 2.15.3.

In order to examine the Algerian population structure, genetic variation was apportioned within and among populations by grouping them according to their North-South geographic regions, and Arab/Berber origins using AMOVA by means of Arlequin software version 3.5 [31]. AMOVA analyses were performed with Y-chromosome and mtDNA haplotypes and haplogroups data independently.

### Autosomal analysis

Autosomal data from three Algerian samples was included in the present analysis. The Zenata Berber sample from Timimoun was newly genotyped with Affymetrix 6.0 array (data available in http://figshare.com/articles/Zenata_genotypes/1534498). The two other populations include the Mozabite Berbers (from the Human Genome Diversity Project, HGDP) and an Arab sample from Algerian general population collected in Algiers [3]. Reference samples from Sub-Saharan Africa (Yoruba), Europe (French Basques) and the Middle East (Palestinians) [32] were used in comparative analyses. The number of individuals per population was randomly subsampled to 20 individuals (except the Algerian Arab sample, which has 19 individuals), thus 119 individuals remained in the analysis. Data from HGDP genotyped with Illumina 650Y was merged with the Algerian data (Arab and Zenata) genotyped with Affymetrix 6.0, thus only SNPs present in both platforms were kept for the analyses. SNPs with different strad assignment in both platforms were removed. After that, populations were individually filtered for missing SNPs and Hardy-Weinberg equilibrium (HWE). HWE deviation was restricted to a p-value > 0.05 and SNPs with missing ratios higher than 0.01 were removed. After the filtering, individual samples with more than 10% missing SNPs were discarded. In order to prevent relatedness, Identity by State (IBS) was checked in order to avoid individuals with similarity greater than 0.85. SNPs with minor allele frequency lower than 0.05 in the whole dataset were removed. Finally, a linkage disequilibrium pruning was performed for a window size of 50 SNPs, a shift step of 5 SNPs and a correlation coefficient threshold of 0.5. Filters were performed with PLINK [33] and applied separately to autosomes and the X-chromosome. A total of 92,744 autosomal SNPs and 3,018 SNPs from the X-chromosome remained for further analyses.

Population structure was investigated with Principal Component Analysis (PCA) plots and ADMIXTURE analysis [34]. PCA was performed with EIGENSOFT 4.2 [35,36] and plots were drawn with R 2.15.3 [37]. ADMIXTURE was performed from with K ranging from 2 to 7 and results were plotted with Distruct1.1 [38]. The autosomal and X-chromosome SNPs were analyzed separately.

The correlation between autosomal and X-chromosome ancestral frequencies found with ADMIXTURE was calculated with R 2.15.3.

## Results

### Uniparental lineages in Algeria

#### Y-chromosome analysis

The paternal lineage composition in Algerian samples is similar to other NW African populations, being E1b1b1b-M81 the most frequent haplogroup, followed by E1b1b1a-M78 and J subgroups [15]. Although the most frequent haplogroup in all Algerian samples is E-M81, the haplogroup frequencies vary among samples (standard deviation = 6.197) regardless their linguistic affiliation (chi-squared test p-value = 0.46). Some sub-Saharan lineages, such as E1b1a-M2, are present at non-negligible frequencies in some samples, such as the Zenata (~23%), whereas some European lineages such as R1-M173 are non-uniformly represented in the present sample set (standard deviation = 16.306). Haplogroup diversity in the Reguibate and the Mozabite was the lowest compared to the other Algerian samples (S2 Table). It is noteworthy that the lowest haplogroup diversity is not related to the current ethnolinguistic affiliation, with some Berber groups such as the Zenata presenting high haplogroup diversities whereas some non-Berber groups such as the Reguibate showing low haplogroup diversity.

**Fig 2:**
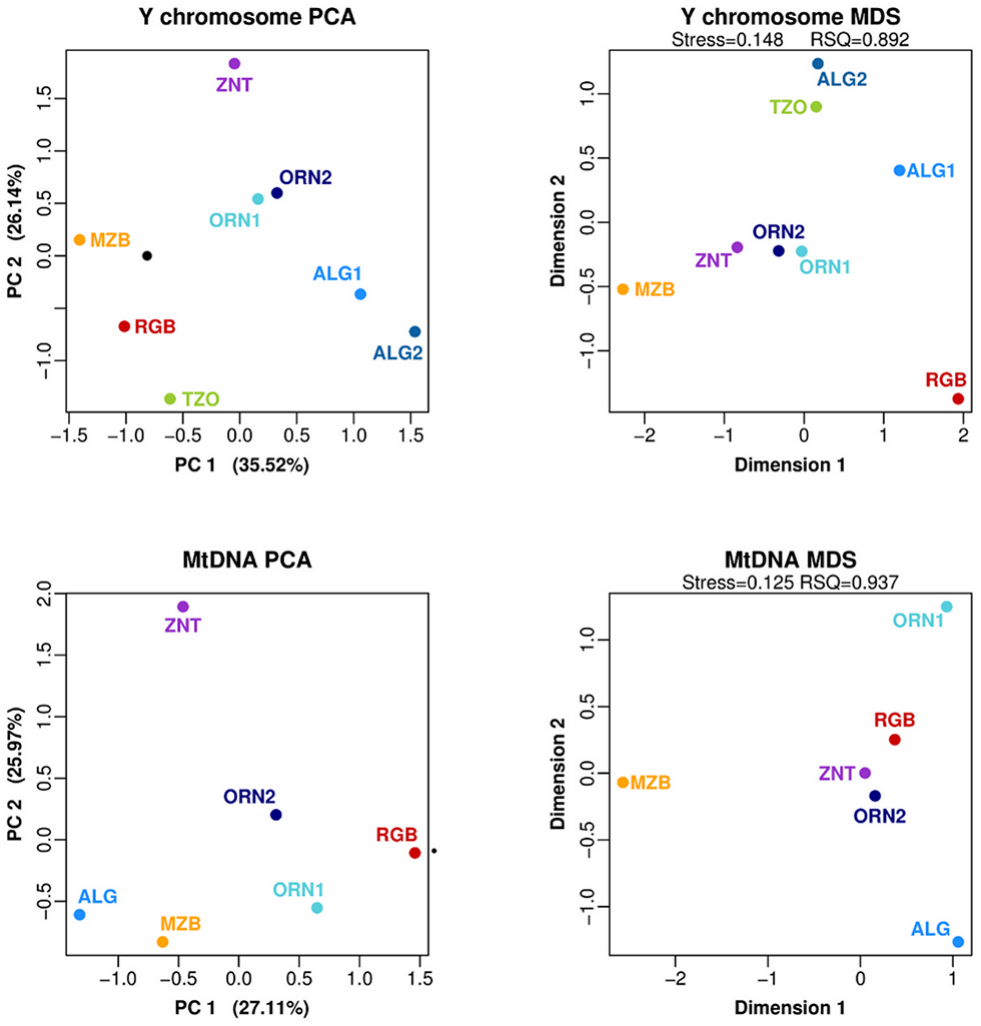
Bidimensional plots based on uniparental genomes. PC analyses based on haplogroup data for Y-chromosome and mtDNA; and MDS analyses based on Y-STR haplotype data and on mtDNA sequence data. Abbreviations: ALG/ALG1: Algiers (this study), ALG2: Algiers (Y-chromosome; [10]), ORN1: Oran (present study), ORN2: Oran (Y-chromosome, [21]; mtDNA, [17]), RGB: Reguibate, ZNT: Zenata, MZB: Mozabite, TZO: Tizi Ouzou

To describe the relationships among Algerian samples, principal component analysis (PCA) and multidimensional scaling (MDS) analyses were performed. The PCA plot performed on the Y-chromosome haplogroup frequencies (Fig 2) shows that both Oran and Algiers samples are clustered together, respectively, and the first PCA component separates the northern populations (Oran and Algiers) from the Mozabite and the Reguibate, which present higher frequencies of haplogroup E1b1b1b-M81 whereas E1b1b1a-M78 is absent in the southern populations. In addition, pairwise genetic distances based on allele size length (R_ST_) were calculated among samples (S3 Table) and significant distances (P<0.0001) were observed between northern populations (Oran and Algiers) and the Reguibate and Mozabite populations, which, in turn, present also significant differences between them. This fact was reflected in the MDS plot in which the first dimension separates Reguibate and Mozabite (Fig 2). Both Algiers and Oran samples show subtle differences in the PCA and MDS plots, which suggest some genetic heterogeneity in Algerian urban areas.

The genetic heterogeneity in paternal lineages in Algeria shown in the previous analyses was demonstrated in an AMOVA when considering all samples together (Table 1). Geographical location of the samples (North or coastal versus South or inland) can partially explain this differentiation since the haplogroup composition show differences between both groups, although no differences were found considering Y-STR haplotypes. No genetic structure was found regarding ethnolinguistic affiliation (Berber versus Arab); however, when Reguibate were removed from their Arab group and considered independently, significant differences were found.

**Table 1:** Y chromosome Analyses of Molecular Variance (AMOVA) in the Algerian samples.

Groups	Among groups		Among populations within groups		Within populations	
	Y-STR	Y-SNP	Y-STR	Y-SNP	Y-STR	Y-SNP
All populations			3.44 ***	4.59 ***	96.56 ***	95.41 ***
North vs South	0.50 ns	5.53 *	3.18 ***	1.64 *	96.33 ***	92.83 ***
Arabs vs Berbers	0.49 ns	-0.50 ns	3.24 ***	4.80 ***	96.27 ***	95.70 ***
Arabs# vs Berbers vs Reguibate	2.62 *	4.94 *	1.78 ***	1.41 ns	95.59 ***	93.65 ***

*** P<0.0001

* P<0.05; ns: not significant

North = Algiers, Oran, Tizi Ouzou

South = Mozabite, Reguibate, Zenata

Arabs = Reguibate, Algiers, Oran

Berbers = Zenata, Mozabite, Tizi Ouzou

^#^ Arabs without the Reguibate

#### Mitochondrial DNA Analysis

An admixture of Eurasian, North African, and sub-Saharan African mtDNA lineages is found in all Algerian samples (S4 Table and S5 Table) as shown in other North African populations [15]. Sub-Saharan lineages were remarkably frequent in the Zenata (L lineages represent ~65%) compared to the rest of the Algerian samples. In particular, West African lineages (such as L1b, L2a, L2b, L2c1, L3b, L3d) add up to over 40% in the Zenata population, but the East African haplogroups (such as L0, L4b2) do not exceed 3.5% in the Zenata or in any of the other Algerian samples. It is also worth to note that the North African mtDNA haplogroup U6 is absent from the Algiers sample and it is only present in one Zenata individual, while it reaches 8.3–28.2% in other Algerian samples. Finally, M1, another North African lineage, is not found in the Zenata sample.

PCA carried on mtDNA haplogroup frequencies (Fig 2) showed that the first component separates the Reguibate (characterized by high frequencies of haplogroup U6a) whereas the Algiers sample lies on the opposite side of the component (with high frequencies of Middle Eastern haplogroup J). The sub-Saharan contribution differentiates the Zenata population from the others in the second component due to the presence of sub-Saharan lineages L2a, L3d and L1b.

Pairwise based F_ST_ distances between the studied populations were calculated (S6 Table) and the genetic affinities between them are reflected on the MDS plot (Fig 2). The highest genetic distance was observed between the Mozabite (F_ST_ = 0.055) and the Northern populations (Algiers and Oran1) (Oran1 is the analyzed Oran sample in this study), whereas, the geographically neighbors Reguibate and Zenata cluster together (F_ST_ = 0.018). The Mantel test shows no correlation between the mtDNA and Y-chromosome Fst vales (p-value = 0.3).

The AMOVA analysis based on mtDNA haplogroups and haplotypes data shows no differences between the Southern and Northern populations and between the Arabs and Berbers, even excluding the Reguibate sample from the Arab group (Table 2). Significant differences are found within populations reflecting their high degree of heterogeneity, which is in agreement with the high genetic diversity observed for both haplogroup and haplotype analyses.

**Table 2:** mtDNA Analyses of Molecular Variance (AMOVA) in the Algerian samples.

Groups	Among groups		Among populations within groups		Within populations	
	mtDNA haplotype	mtDNA haplogroup	mtDNA haplotype	mtDNA haplogroup	mtDNA haplotype	mtDNA haplogroup
All populations			3.23 ***	2.09 ***	96.77 ***	97.91 ***
North vs South	-0.15 ns	-0.10 ns	3.32 ***	2.15 ***	96.82 ***	97.95 ***
Arabs vs Berbers	0.19 ns	0.92 ns	3.14 ***	1.65 ***	96.67 ***	97.43 ***
Arabs# vs Berbers vs Reguibate	-0.27 ns	0.16 ns	3.43 ***	1.98 ***	96.84 ***	97.87 ***

*** P<0.0001; ns: not significant

North = Algiers, Oran (both)

South = Mozabite, Reguibate, Zenata

Arabs = Reguibate, Algiers, Oran1, Oran2

Berbers = Zenata, Mozabite

^#^ Arabs without the Reguibate

### Genome-wide data analysis in Algerian samples

**Fig 3:**
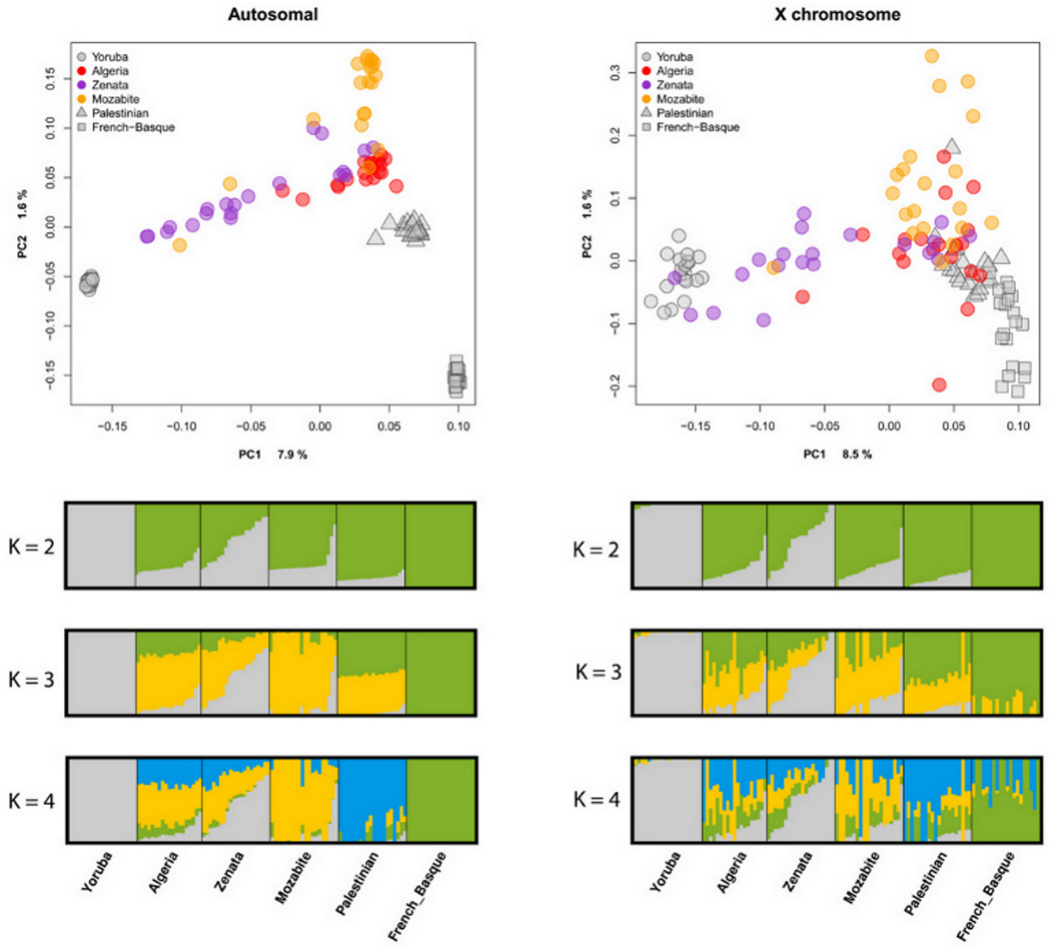
Plots for the analysis of genome-wide SNPs. PC analysis (upper figures) based on autosomal data, and X-chromosome SNPs. ADMIXTURE proportions (bottom figures) at k = 2,3, and 4 based on autosomal data and X-chromosome SNPs. Algeria, stands for general Algerian sample [3]; Mozabite, stands for the Algerian Berber Mozabites [32]; and Zenata, stands for Algerian Berber Zenata (present study).

A genome-wide SNP array typed in a subset of individuals and samples was analyzed to complement our knowledge of the genetic structure in the Algerian population. The first two axes of the PCA show a differentiation of sub-Saharan (i.e. Yoruba), Middle Eastern (i.e. Palestinian), European (i.e. Basque), and Algerian individuals (Fig 3). All individuals form compact sample clusters with the exception of Algerian Berbers (Mozabite and Zenata) who are spread in the plot showing a gradient towards sub-Saharan Africans while others remain closer to the Algerian non-Berber sample. Moreover, Fst values reveal a higher similarity of the Zenata population with Yoruba than any of the other North-Africans (Fst = 0.037) (S7 Table). Interestingly, most of the Mozabites form a cluster separated from the Algerian non-Berber individuals at one of the edges of the second PCA.

In order to establish putative ancestral components in the genotyped individuals, an ADMIXTURE analysis was performed for the same samples (Fig 3 and S1 Fig). At K = 2, one ancestral component is associated with sub-Saharan individuals while the other one is associated with the Europeans, showing the rest of individuals an admixture of both components. At K = 3 a North African/Middle Eastern ancestral component appears, whereas at K = 4 a Middle Eastern ancestral component arises (higher values of K show population substructuring; data not shown). The Algerian (Berber and non-Berber) samples present a complex amalgam of components as found previously in North African populations [3]. The Mozabites show the highest North African ancestry, as expected from its position in the PCA, and also contain very low admixture with Middle Eastern, European or sub-Saharan ancestral populations. In contrast, the Zenata individuals present high variation due to differential sub-Saharan admixture, in agreement with the results shown in the PCA. The North African component in this Zenata sample is not as frequent as in the Mozabites (the mean frequencies in the populations are 0.348 and 0.823 respectively), and the former also contain more admixture from the Middle East.

**Fig 4:**
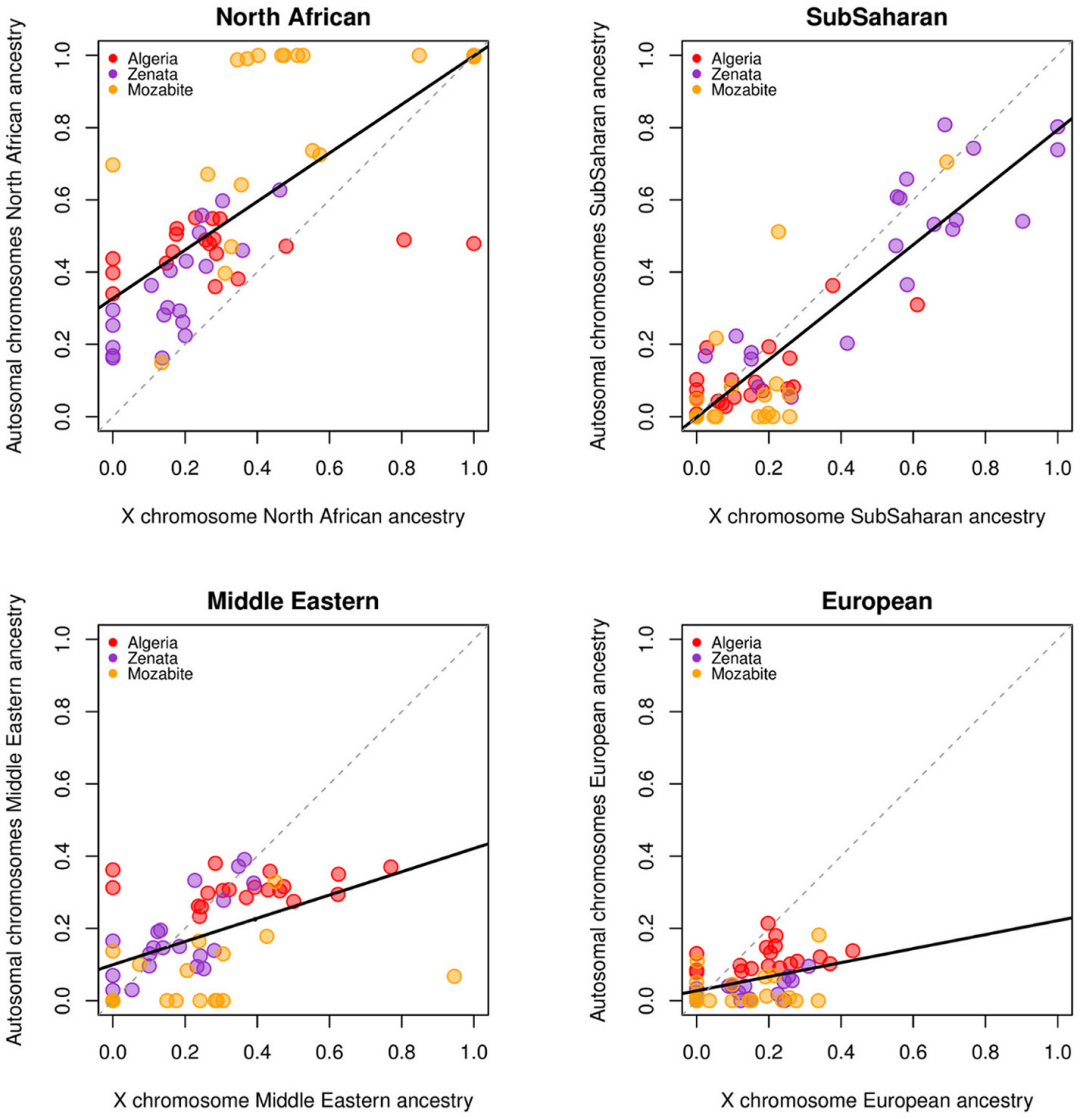
Correlation plots of the ancestry proportions at k = 4 in the ADMIXTURE analysis comparing autosomes and X-chromosome SNPs. North African, sub-Saharan, Middle Eastern, and European ancestry proportions are shown in different plots. Solid black lines represent linear correlations between autosomal and X-chromosome components.

In order to test whether the heterogeneity detected in the Algerian population might be attributed to differential sexual admixture, an analysis of the autosomal versus the X-chromosome diversity was also performed. The results of the PCA and ADMIXTURE analyses carried out exclusively for X-chromosome markers (Fig 3 and S1 Fig) are similar to the autosomal analysis, although the resolution is lower due to the lower amount of SNPs available in the X-chromosome (~3,000 SNPs). Nevertheless, some differences in autosomal and X-chromosome ancestry distribution can be found. North African ancestry is higher in autosomal chromosomes than in the X-chromosome (p-value = 7.73e-11), which could be explained by a contribution of this ancestry mainly driven by men. On the other hand, Middle Eastern, European and Sub-Saharan ancestries might have mostly been driven by women, as shown by the higher frequency of these ancestries in the X-chromosome when compared to autosomes (p-values = 0.004,1.709e-08 and 0.001 respectively) (Fig 4).

## Discussion

Our results of uniparental and autosomal markers in Algeria agree with the presence of ancestral components previously described in North Africa, attesting the genome complexity of Algerians (S8 Table). Concerning the Y-chromosome data, the highest frequencies are seen for the autochthonous North African lineages E-M81 and E-M78, this last one more frequent in Northeastern Africa where it has probably emerged [39]; whereas the presence of the Middle Eastern Y-chromosome J1-M267 has been attributed to the Islamic expansion [40]. In a similar way, the mitochondrial DNA analysis shows also different lineages in Algeria, already observed in North Africa: the North African lineages U6 and M1 that have been dated to Paleolithic times [41–43], the Eurasian H (related to the Neolithic expansion) and HV [29,44,45]; and the sub-Saharan lineages (L). It has been suggested that the sub-Saharan lineages for both mtDNA and Y-chromosome reached very recently North Africa through the slave trade routes across the Sahara [16,46,47]. Moreover, the autosomal genome-wide SNPs analysis also demonstrates the admixture of the Eurasian and African components in both Berber (Mozabite and Zenata) and non-Berber populations from Algeria in agreement with the general genetic North African landscape [3].

The genetic structure observed in the Algerian analyzed groups is neither fully correlated with ethnic affiliation (Berber-Arab) nor with geography (coast vs. inland). It is noteworthy, however, that when we removed the Reguibate population from the Arab group, a significant differentiation was observed between the paternal gene pool profiles of Arabs and Berbers. This absence of differentiation between Arabs and Berbers is in agreement with what has been already observed in several North African populations by the analysis of different genetic markers, such as autosomal classical markers (such as HLA markers and GM allotypes) [6,12,14]; autosomal STRs [7]; Alu polymorphisms [8]; Y-chromosome [13,48]; and mtDNA analyses [11,12]. As a result, it has been suggested that the Arabization of North African populations was mainly a cultural process rather than a demographic replacement of autochthonous groups [11].

The genetic heterogeneity among Algerian populations highlights the complex relations between biological, social, cultural and geographical contexts. The distribution and frequencies of the North African, Eurasian and sub-Saharan components both in uniparental and autosomal markers is variable in each group, not only when comparing Berber and non-Berber, but also within linguistic groups. For example, some autochthonous North African haplogroups were not present in certain samples, such as the mtDNA U6 haplogroup that was absent in Algiers and present in only one individual in the analyzed Zenata group. In the same way, mtDNA haplogroup M1 is absent in the Zenata population. The absence of the maternal North African component in these groups, especially the Zenata Berbers, might be explained by extensive genetic drift and the remarkable high frequency of sub-Saharan lineages (~23% for the Y-chromosome E-M2 haplogroup and ~ 65% of mtDNA L lineages) in the Zenata sample. Our autosomal analysis also shows the close position of the Zenata group to the sub-Saharan populations, and the high variance in this sub-Saharan ancestry suggest that this group has experienced recent gene flow.

The complex demography of the Algerian samples analyzed is also reflected in the sexual bias of gene flow and admixture. MtDNA sub-Saharan haplogroups are more frequent than the Y-chromosome lineages, suggesting higher sub-Saharan female gene flow in our Algerian samples. This is in agreement with autosomal and X- chromosome ancestral components, where there is also evidence of sexual bias in the sub-Saharan component. On the other hand, Y-chromosome results show higher frequency of North African than mtDNA haplogroups. This is in agreement with the results for autosomal and X-chromosome markers, which associate North African component to males, while the Middle Eastern and European ancestries seem to be derived from a female gene flow into Algeria. This difference can be explained by the historic and prehistoric role and status of women in the Algerian society: sometimes considered as an object of warlike conflict or as alliances between occupant's and occupied various clans, as denounced by some ethnologists and historians [49,50].

It has been shown that some Berber populations (Tuareg, Mozabite and Chenini-Douirat) are heterogeneous and outliers within the genetic North African landscape, and they seem to have experienced long periods of genetic isolation without subsequent admixture with other groups [15]. This process of isolation was probably recent and has been followed by genetic drift [11]. However, our results in Algeria challenge the identification of Berber-speaking groups as isolated populations, whereas Arab-speaking groups are identified as genetically more diverse and less isolated. Our results demonstrate that Berber groups are not systematically isolated and closed, such as the Zenata who show a different genetic profile compared to the Mozabites, already known to be an isolated Berber group [18]. Their different genetic profiles reflect probably the notion of an open versus close lifestyle towards the outsiders in their so-called isolated populations. Although the Mozabites are descendants of the Zenata Berber group in North Africa, nowadays, the majority of the Mozabites form an isolated Ibadi Muslim group in Algeria. The Ibadi form of Islam evolved from the 7th century Islamic group known as the Kharijites in Irak. They reached Algeria and found a refuge within the isolated group of the Mozabites [51,52]. Although both Zenata and Mozabite Berber groups are geographically close, their different genetic profiles suggest that Mozabites have been more isolated and received less gene flow than the Zenata, who show more admixture not only with sub-Saharan but also with Middle Eastern populations when analyzing autosomal markers. Although the Zenata was the major Berber group in North Africa, their presence in Algeria in present days is restricted to the city of Timimoun, which has been known by its slave population called the Haratines, dark-skinned people, who lived with the Zenata in the ksours of the Gourara (Timimoun region) and learned from them the Berber language and became freed Muslims [53]. On the other hand, Arab groups can be isolated, such as the present example of the Reguibate that shows the lowest paternal haplogroup diversity with the Mozabites. The Reguibate population might have experienced some genetic drift or a genetic founder effect that altered its unilinear lineage frequencies. Indeed, the Reguibate show the highest frequency of the North African component for both Y chromosome (E-M81) and mtDNA (U6a), after the Mozabite.

It has been observed that cultural isolation in rural communities promotes, by the effect of genetic drift, stronger loss of diversity and larger genetic differentiation levels than those observed in urban areas [54]. In addition, a recent study on the Sousse population from Tunisia has shown that cities represent an opportunity to examine the impact of multiple migrations into a region over the time [55]. Therefore, our results suggest that cosmopolite cities (such as Oran and Algiers) are representative of admixed populations that were subject not only to multiple prehistorically and historical migrations but also to those from the rural communities that continue to present day. Consequently, their genetic diversity is more often increased, which decrease the genetic structure among them, regardless the linguistic affiliation of the individuals.

The overall ancestral proportion of admixture components within populations considering mitochondrial and Y-chromosome haplogroups and autosomal markers reflects a similar history of gene flow at the population level. However, the comparison of the different genetic markers at individual level reflects differences as a result of the difference inheritance models of each marker. This discrepancy can be seen in present example of the Zenata sample where these markers were tested in each individual (S2 Fig). It is clearly shown that there is no correlation between the ancestral component origin of the mitochondrial and the Y-chromosome haplogroup in each individual. For example, some individuals show a typical sub-Saharan maternal haplogroup and a North African paternal one. Autosomal analysis can also provide different distribution of ancestral components that is not related to the origin of the uniparental haplogroups. The analysis of different regions of our genome might provide different insides in the population history of the samples under study, thus allowing a wider combining vision of the ancestral histories stored in each marker.

## Supporting Information

S1 Fig ADMIXTURE cross-validation errors and log-likelihoods for the autosomal and X-chromosome SNPs. (TIF)

S2 Fig Individual ancestry components obtained by ADMIXTURE and uniparental haplogroups geographically sorted of the genotyped Zenata individuals. (TIF)

S1 Table Y-chromosome haplogroups and STR haplotypes in the analyzed Algerian samples. (XLSX)

S2 Table Y-chromosome haplogroup frequencies among the studied populations (% in parentheses). (XLSX)

S3 Table Pairwise genetic distances (R_ST_) calculated using the 10 Y-STRs (DYS389I, DYS390, DYS389II, DYS19, DYS393, DYS391, DYS439, DYS392, DYS437, DYS438). (XLSX)

S4 Table mtDNA analysis detailed results of the studied samples. (XLSX)

S5 Table mtDNA haplogroup frequencies (%) distribution among Algerian populations. (XLSX)

S6 Table Pairwise based F_ST_ distances between the studied populations based on mtDNA sequences analysis. (XLSX)

S7 Table Autosomal Fst values for the studied populations. (XLSX)

S8 Table Summary of the mean percentages of the ancestral populations in each genomic region. (XLSX)
