# Supplementary figures and images for: Genetic Heterogeneity in Algerian Human Populations
Source: PLoS One. 2015 Sep 24;10(9):e0138453. doi: 10.1371/journal.pone.0138453 (PMC4581715; doi:10.1371/journal.pone.0138453)

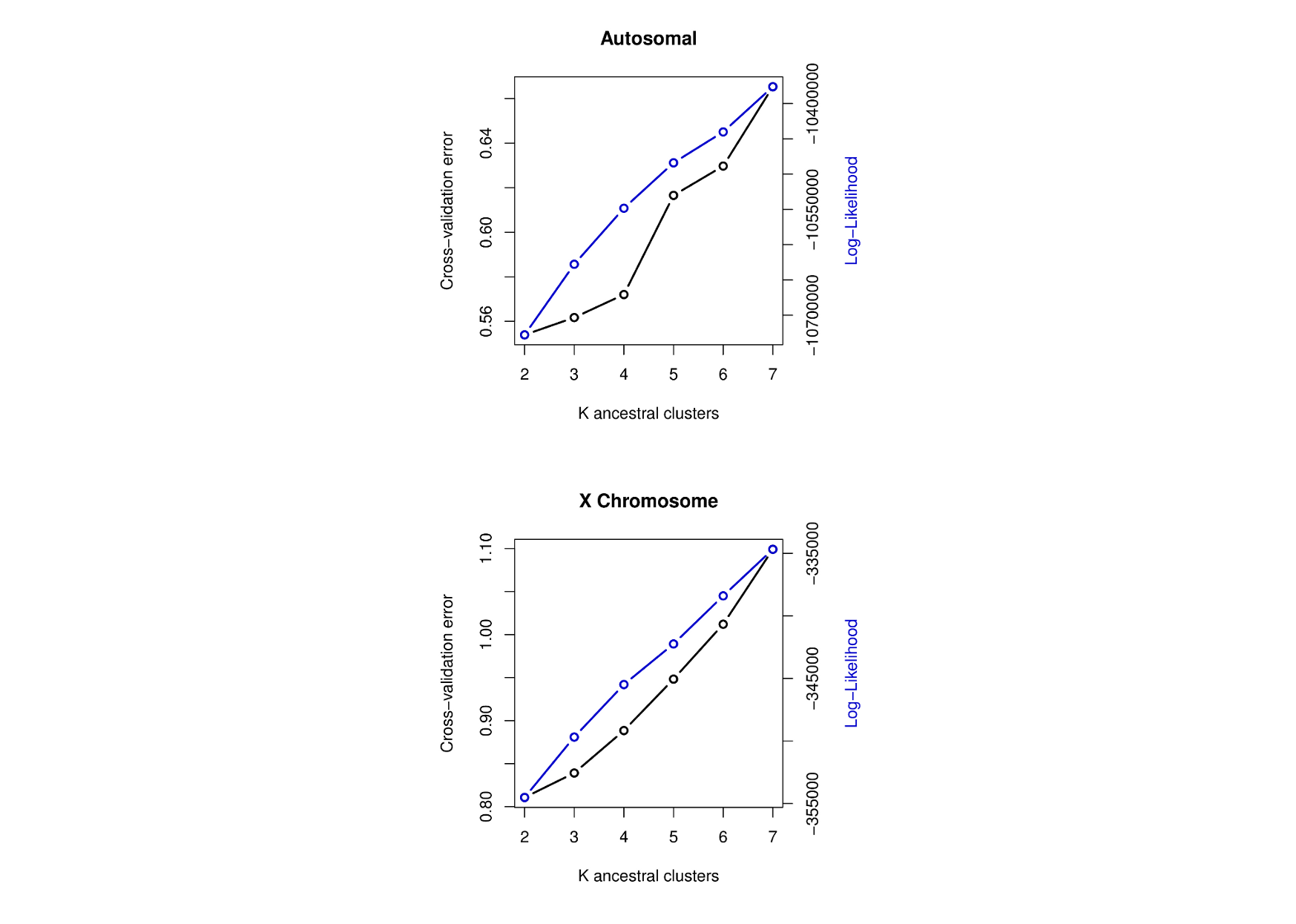

Supplement: S1 Fig — (TIF) [file pone.0138453.s001.tif]

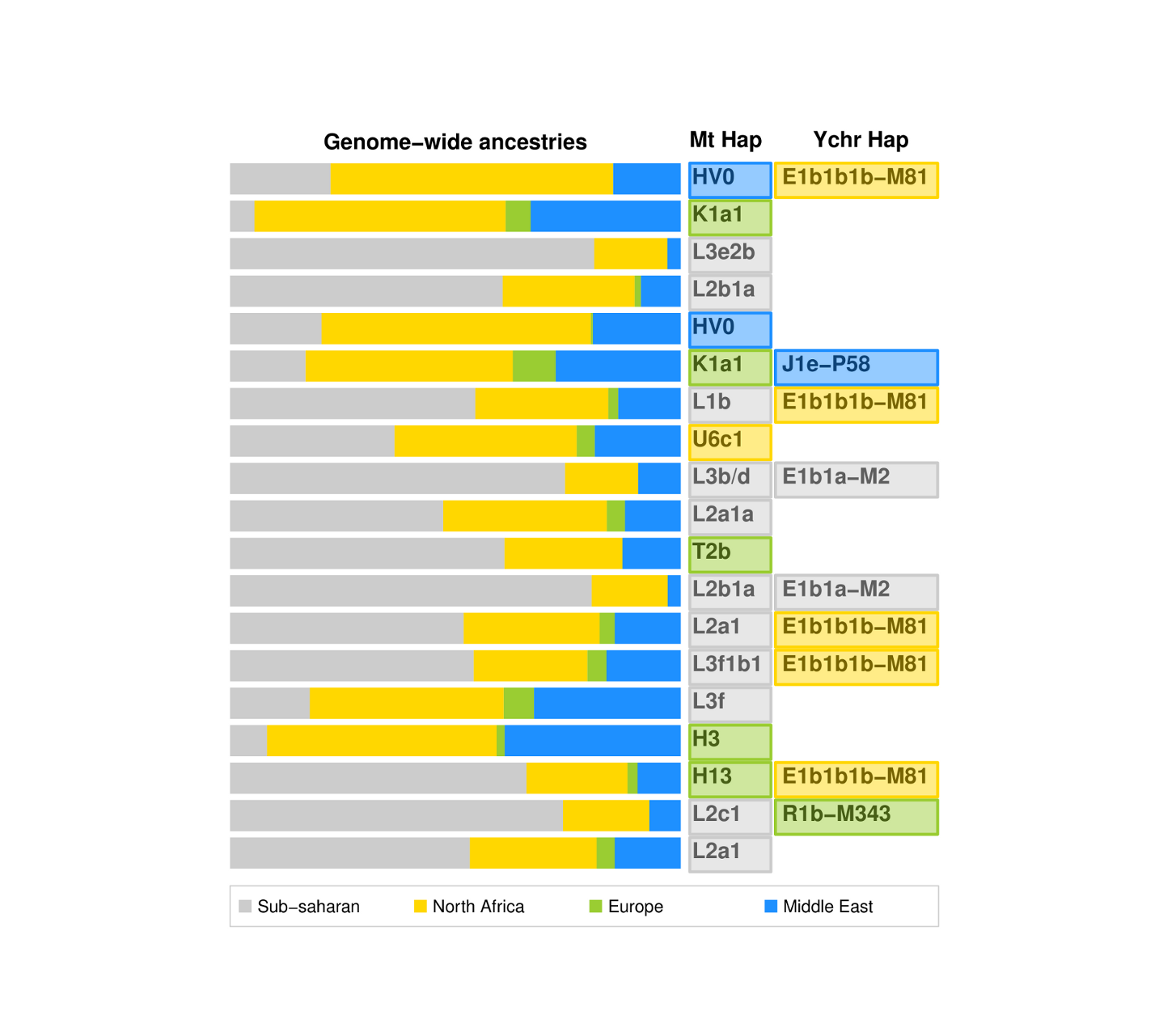

Supplement: S2 Fig — (TIF) [file pone.0138453.s002.tif]
